# Notes from Self-Isolation: Imagination in Times of Ruptures

**DOI:** 10.1007/s12124-025-09904-9

**Published:** 2025-09-22

**Authors:** Hana Hawlina, Tania Zittoun

**Affiliations:** 1 Institute of Criminology at the Faculty of Law Ljubljana, Poljanski Nasip 2, 1000 Ljubljana, Slovenia; 2 Department of Psychology, University of Ljubljana, Aškerčeva Cesta 2, 1000 Ljubljana, Slovenia; 3 Institute of Psychology and Education, University of Neuchâtel, Espace Tilo-Frey 1, 2000 Neuchâtel, Switzerland

**Keywords:** Covid-19, Imagination, Rupture, Collective diary, Sense-making, Symbolic resources

## Abstract

The Covid-19 pandemic brought about an unprecedented global health crisis, which caused a seismic disruption of people’s lives, their habitual practices, systems of meanings, and relationship to the past and the future. This contribution will explore how a group of 17 participants who wrote a collective diary during the first wave of the Covid-19 pandemic in Slovenia (March–May 2020) experienced the crisis as a personal and collective rupture, and what were the functions of the imagination in managing uncertainty, constructing new meanings, and ultimately adapting to the novel situation. Based on the diary data, we find that the global pandemic crisis was experienced as a rupture along four central dimensions: temporality, spatiality, sociality, and embodiment. Drawing on the conceptualisation of the imagination in sociocultural psychology, we have identified the functions of the imagination in different stages of adaptation to the rupture (e.g., experiencing the rupture, meaning-making, distanciation, symbolic mobility, temporal projection), and observed how people use symbolic resources to make sense of the situation, cope with the uncertainty, and construct new imaginings of the future. We thus posit that the imagination plays a central role in repairing ruptures, both in terms of semantic reconfiguration and guiding future-oriented action.

## Introduction

The COVID-19 pandemic brought about a global health crisis, which dramatically disrupted the habitual practices of millions of individuals, institutions, and entire systems (e.g., health, economic, manufacturing, transportation). During the first wave, people were confronted with a new situation; public discourses called it “unprecedented times”, reflecting a rupture that people experienced in their daily lives. Our study seeks to explore a fundamental question of how people react to such ruptures and in what ways does imagination contribute to making sense of the unfamiliar situation and reconstructing a sense of normality. Since the current era is increasingly characterised by crises to the extent that the state of the world is often described as a "polycrisis" (the convergence and mutual amplification of multiple crises; Henig & Knight, 2023; Lawrence et al., 2024), it is crucial to better understand the personal and collective experiencing and coping in difficult times.

The COVID-19 outbreak was first identified in China in November 2019, from where it rapidly spread to the rest of the world. The World Health Organization officially recognised it as a pandemic on March 11th, 2020 (Cucinotta & Vanelli, 2020). In a matter of merely weeks, it led to the creation of new social norms, prescribed and restricted behaviours, generated fears and anxieties, called for the creation of systems of information (and misinformation), etc. – the pandemic created an entirely new social reality (Karwowski et al., 2021). Globally, hundreds of millions of people have been infected, and the SARS-CoV-2 virus has claimed over 7 million lives worldwide by early 2025 (World Health Organization, 2025). The pandemic has destabilised national economies, the effects of which are still debated (Pangallo et al., 2024; Rathnayaka et al., 2022) and exacerbated the already existing economic, educational, and ethnic inequalities around the world (Armitage & Nellums, 2020; Beaunoyer et al., 2020; Burström & Tao, 2020; Fisher & Ryan, 2021; Pellicano & Stears, 2020; Van Dorn et al., 2020).

We will focus on the first wave of the pandemic in Slovenia, during which the restrictive measures imposed by the national government to contain the SARS-CoV-2 virus disrupted people’s organisation of everyday life, and asked them to cope with social distancing and self-isolation as public life was suddenly entirely relocated from shared spaces to the online spheres (Ružić Gorenjec et al., 2021). A substantial number of habitual practices, personal routines, and social rituals became unavailable and inaccessible, as most of people’s activities were reduced to the private sphere of one’s home.

The central question of this paper is, how do people deal with such societal crises, what are the ruptures they experience, and how, through imagination, do they make sense of the situation and reconstruct a sense of normality? By observing a group of people who wrote diary entries during the two-month period of self-isolating that characterised the first wave of the COVID-19 pandemic in Slovenia, we will explore how they made sense of a novel social reality in the absence of collective guidance. We will show that crises disrupt people’s sense of time, space, social relations and even relation to their bodies; we argue that imagination plays a major role in making sense of these ruptures, and put to the fore the evolving functions of the imagination in repairing these ruptures.

### The Pandemic as Unprecedented Crises

During the first months of the pandemic, political speeches, everyday greetings, as well as formal and semi-formal emails often began with the address “In these unprecedented times” (Francis, 2020; Sauer, 2020; Saunders, 2020). This phrase has become an unofficial slogan of the COVID-19 pandemic,^1^ since it captured the personal and collective experience of a seismic disruption. It is very illustrative of the collective uncertainty that marked the first wave of the pandemic – it encapsulated the shared sentiment of living through a global crisis, which felt deeply unfamiliar, and which has disrupted the habitual temporality and created a novel, ruptured experience of time. In the next section, we first introduce how we apprehend crises and their consequences in ruptures from the perspective of sociocultural psychology; second, we show how imagination may play a specific role in overcoming ruptures.

### Crises, Ruptures and Imagination

The COVID-19 pandemic can be considered as a global crisis (Koselleck, 2006): the virus caused a global period of disruption of routines, meanings and behaviours for billions of people. Almost every element of the COVID-19 outbreak was questioned—its origins, extent, infectivity, durability, and lethality (Durodié, 2020). Thus, the people could not establish a stable set of meanings and solutions, but engaged in an ongoing process of constructing meanings of the evolving situation, which was not only made possible, but necessary by the COVID-19 pandemic (Durodié, 2020; Latour, 2020). In that sense, COVID-19 created a collective crisis.

Collective crises are not only social, political or public health issues; they create a semantic disruption of the stable, taken-for-granted meanings, destabilise people’s quotidian practices and temporal organisation, and affect their future life course trajectories. Such crises have been studied by social psychologists, who explored the role of social representations in enabling communities to turn the unfamiliar into the familiar (Moscovici, 2000, 2008). If crises call for collective meaning-making, these are also experienced more personally as ruptures, which call for individual sense-making. In sociocultural psychology, a *rupture* is conceptualised as an event that creates a break, or discontinuity in a person’s experience and questions what they hold as taken for granted (Hviid & Villadsen, 2015; Zittoun, 2016, 2021; Zittoun & Gillespie, 2012; Zittoun et al., 2003). To repair the ruptures, people engage in sense-making, which involves giving a semiotic form to experiences, articulating them in relation to other experiences, and projecting them through time (Salvatore et al., 2008; Zittoun, 2011, 2017). Studies have thus shown the role of narratives (Bruner, 2003; Daiute, 2010), dialogical dynamics, and uses of symbolic resources in making sense of and repairing ruptures (Gillespie et al., 2008; Kadianaki, 2014; Märtsin, 2019; Zittoun, 2021; Zittoun et al., 2008). More specifically, we posit that the imagination, which can be supported by all these processes, may play a particular role in dealing with experienced ruptures (Zittoun & Gillespie, 2016; Zittoun, 2018a).

Sociocultural psychology understands the imagination as a process as well as a higher mental function that is dynamic and highly contextual, both nourished and constrained by culture and its various social, material, and symbolic resources (Vygotsky, 2004; Zittoun & Gillespie, 2016; Zittoun et al., 2021). Imagination can be conceived of as a loop, a process through which the flow of consciousness temporarily disengages from the immediate situation and the sensorial input of the physical reality to explore alternative possibilities. Sequences of imagining are usually *triggered* by specific ruptures or dissatisfaction with the present situation; imagination is then nourished by various *resources* on which the person or groups can draw and creatively recombine (personal experience, culturally shared meaning complexes, symbolic resources such as books, films, narratives), and have a variety of *outcomes,* from a slight shift in mood to writing a novel or creating a new perspective on an unfamiliar situation. All in all, imagination can substantially expand one’s experience (Zittoun & Cerchia, 2013; Zittoun & Gillespie, 2016).

If it is established that imagination can be triggered by events which are experienced as ruptures, and that it enables exploring alternatives in points of bifurcation (Zittoun & Valsiner, 2016), its role and dynamics in times of collective crises have yet to be more systematically explored. Social crises such as the refugee or migration crises have been theorised in relation to social imaginaries (Appadurai, 1996; Salazar, 2011), but a framework of how these collective dynamics are connected to more personal imagination and sense-making has remained underdeveloped (Zittoun, 2020a). This is what we propose in this paper; we consider how a group of persons experiencing the same collective crisis, which generated ruptures in each of their lives, engaged in imagining as they tried to make sense of the situation.

In summary, collective crises cause ruptures in people’s trajectories; ruptures call for sense-making and the work of imagination; thus, ruptures can act as a catalyst for personal and social change (Hawlina et al., 2020; Kadianaki & Gillespie, 2015; Markova, 2008; Zittoun, 2018b). We aim to explore what makes the COVID-19 crisis specific; why was it perceived as “unprecedented”? Concretely, which ruptures did people experience? How did people make sense of them, and what role did imagination play in their sense-making?

### Defining Ruptures in Unprecedented Times – the Collective Diary

Although the COVID-19 was a pandemic affecting the whole world, people’s experiences were modulated by their national policies. In Slovenia, like in most European countries, a forced confinement was imposed: without any preparation, people were asked to remain home, transfer their usual activities – education, work, leisure – to their place of residence, and were asked not to meet people out of the household to avoid the virus from spreading (Petković, 2020; Plavčak, 2020; Trampuš, 2020). In that respect, people did not only have to make sense of bad news, or a distinct social representation; they had to drastically adjust to extremely new and unexpected living conditions by changing their daily lives.

In this paper, we show the activity of meaning-making engaged by a group of people in Slovenia who spontaneously began to write a collective diary. Like any diary, a collective diary enables following people’s day-to-day lives, as they try to make sense of the events and experiences of the day (Zittoun & Gillespie, 2022). People make sense in the very act of writing, addressed to an imaginary or real audience. Here, as people wrote an online, collective diary, they were thus engaged in a dialogical and collective practice of meaning-making, reading each other's entries before adding their own.

Based on our analysis of the diary data, the emerging scientific literature and the broader discourses around the pandemic, we propose to characterise the rupture triggered by the SARS-CoV-2 pandemic along four main dimensions, which confer its specificity (see Analysis for how the dimensions were identified). We will argue that it affected people’s sense of temporality, the experience of spatiality, their sociality, and that it had a specific embodied dimension. The dimensions are intersecting and interdependent. In order to repair the rupture, people have to imagine and make sense along each dimension, using whatever resources were available. Here, we briefly announce the dimensions of the ruptures along which sense-making was constructed via imagination; the subsequent analysis will be organised around them.

- Temporality

The COVID-19 pandemic was initially seen as a temporary departure from the ordinary state of affairs and an interim period of exception between a pre-pandemic and post-pandemic “normality”. However, it quickly became clear that it was more than an interim. Hence, as we will see, one of the most prominent characteristics of the pandemic was the collapse of the shared time markers and social organisation of time; for many people, the shift to self-isolation and/or working from home meant that time became undifferentiated, without clearly bounded markers (such as the beginning and the end of the workday, scheduled daily and weekly activities, a clear difference between weekdays and weekends, etc.). Furthermore, many cultural events (such as the Eurovision, Olympic games and other sporting events, tentpole film releases, etc.) were cancelled or postponed, and, in contrast with other disrupted times (Zittoun, 2008), even holidays lost a great deal of their social and cultural significance when the collective rituals (such as gathering and painting eggs during Easter) became inaccessible, and there was little differentiation between holidays and ordinary working days. In the absence of the habitual structuring of time, people had to create new semiotic coordinates and private time markers to navigate the otherwise undifferentiated days in confinement, and to accord meaning to the pandemic period. However, imagination precisely enables one to travel in time, from past to future and back (Zittoun & Gillespie, 2016); since it is freed from temporal succession, it is interesting to explore how imagination could repair sense-making beyond temporal ruptures.

b) Spatiality

A second specificity of the rupture engendered by the COVID-19 pandemic was the physical immobility it imposed, due to the prescriptions to isolate oneself within one’s home, often accompanied by the restrictions on travelling across municipal, regional, and national borders Restricted mobility and sociality can be experienced by people relocating to more or less total institutions such as a nursing home (Zittoun et al., 2021), a psychiatric unit (Brown & Reavey, 2016; Kanyeredzi et al., 2019), or even a prison (Gök & Kara, 2021; Gregoriou, 2021). However, unlike in the case of prison or institutionalisation, which usually affects some group of people only (for political, disciplinary, health reasons, etc.), the COVID-19 imposed immobility and indiscriminately forced confinement to almost everyone, except for essential workers.

The restricted geographic mobility during self-isolation meant that diverse spheres of experiences and activities were conflated to the confinement of one’s home. The lack of spatial diversity and spatial boundaries corresponded with the lack of temporal boundaries, which led many people to feel an overwhelming sense of sameness from day to day (Cangià, 2017). Hence, unlike other crises, which normally brings some groups of people to more mobility than others, here people were almost all, massively and indiscriminately, required to become immobile (but see e.g. (Piccoli et al., 2021). However, in case of forced immobility, people may imagine distant place and spaces, thus generating alternative modalities of symbolic mobilities (Zittoun, 2020b); how was this possible here, and how much did it support the repair of the disruption of the sense of space?

c) Sociality

The onset of the COVID-19 pandemic was characterised by a lack of stable social guidance: there was an absence of coherent narratives about the causes of the crisis as well as its possible future progression, people were dealing with delayed official responses, an abundance of misinformation, and the creation of many politically-motivated false narratives surrounding the virus (Cuan-Baltazar et al., 2020; Evanega et al., 2020; Gupta et al., 2020; Ricard & Medeiros, 2020). Such an absence of a social consensus and culturally available resources demanded a great deal of collective and individual meaning-making and creative re-combining of cultural resources to make the situation legible, as is the case with the emergence of any unfamiliar, destabilising novelty (Emiliani et al., 2020; Jovchelovitch, 2019; Moscovici, 1981).

Furthermore, unlike in most other collective crises, the pandemic brought about an unprecedented set of restrictions on social contact in the interconnected, globalised world of the twenty-first century. The preventive measures of social distancing meant that face-to-face interactions were severely limited to only the closest members of family and household, while the majority of social life was relocated to online spaces, mediated via video conference programmes and social networks. How was it then possible to imagine the others in absence, how could people restore a sense of sociality through their imagination?

d) Embodied dimension

Finally, the SARS-CoV-2 virus presented a very real, physical threat to people’s health and wellbeing. The body was reimagined as a vulnerable site of possible infection, and the space beyond the boundaries of the body was reimagined as dangerous and potentially full of invisible airborne viruses. The new meanings allocated to bodies as vulnerable and at a constant risk of infection corresponded to the emergence of new behavioural practices and routines of hygiene and prevention, as well as led to the attempt to create of new social categories of “vulnerable” groups of people (Hurst et al., 2020). Such social categorisation and stigmatisation in the face of an epidemic characterised the AIDS crisis of the 1980s (Joffe, 1995, 1998), however, unlike other crises, it soon occurred that everyone was likely to be vulnerable. If imagination is also partly embodied (Gfeller, 2017; Gfeller & Zittoun, 2020), how much could people re-imagine these bodies in disrupted times and make sense of them?

In short, in what follows, we examine the ways in which a group of people made sense of the temporal, social, spatial and embodied ruptures experienced while they were facing the crises that was the first wave of the COVID-19, and show how imagination, individually and collectively, supported such a process.

## Methodology

In what follows, we present the data on which this paper is based, a collective diary, and the methods with which it was analysed.

### The Collective Diary

At the beginning of the pandemic in Slovenia, a group of 17 adults (aged 21–64 years; 14 female, 3 male) decided to write a collective diary of the time in self-isolation. The diary was started on March 15th, 2020, and concluded on May 10th, 2020, when the period of highly restrictive measurements and self-quarantine had come to an end. During this time, most of them lived in apartments in the capital city, Ljubljana, and studied or worked in professions that allowed them to comply with self-isolating measures.

The initiative to write the diary began in a group chat between March 14th and 15th, when the pandemic had just been declared in Slovenia, and the participants faced a highly novel and uncertain situation, which they perceived as “living in a historic time” (quote from the chat). The idea to chronicle this period in a collective diary emerged on a WhatsApp group created to keep up with friends at the onset of the pandemic, make sure that everyone is safe, exchange news, and cheer each other on. One of the group members started a shared Google Document, where everyone could contribute their entries, and titled it “Notes from Self-Isolation” (in reference to Dostoevsky’s *Notes from Underground*); thus, a collective diary was born. Over the eight-week period, the participants produced 324 entries, which included text and images. There was no pre-determined or suggested topic – the participants wrote whatever they perceived as meaningful and significant, and narrated their experience of self-isolation in their own words, curating what to include in their entries. Due to the scope of this paper, the images have been excluded from the analysis; in most cases, they serve to support and further illustrate what the diarist wrote about (e.g., if they remembered a concert they attended before the social distancing came into effect, they would add a photo from the concert).

From the beginning, the participants imagined the collective diary as a future historical document; the diary was later anonymised and submitted to the archive of the Ljubljana City Museum, which issued a call for documents of the pandemic period in Slovenia. The diary was entirely participant-driven, with no instructions, predefined structure, or external incentives. Contributions emerged organically in response to the group's evolving needs and ceased when participants no longer felt compelled to write. Unlike solicited personal writing in research, this diary was a spontaneous, collective endeavour. The first author was one of the diarists, and knew most of the others, since she was one of the members of the group chat where the initiative started. She participated without analytical intent, only later recognising its research potential. As such, no a priori asymmetry existed between the researcher and participants – the first author contributed to the diary as a peer, sharing the same motivations for writing. This emic perspective facilitated a deeper understanding of the data but also called for reflexivity on the author's dual role of researcher and participant. None of the author's own entries were included in this paper, and the fidelity of the analysis and interpretations was subsequently discussed with other participants.

Although diary methods have a long tradition in autoethnography, given that self-writing is a useful tool for developing the researcher’s reflexivity and that it offers experiential insight into the studied phenomenon (Choi, 2016; Wall, 2008; Winkler, 2018), research on collective diaries is still scarce (Mackenzie et al., 2013; Matsui, 2002). In terms of the digital medium, a wider audience and sense-making function, a collective diary can be compared to public online diaries and blogs (Gillespie et al., 2024; Mark et al., 2012), but it differs in terms of who the audience is and the addition of textual interactions and cross-references among the writers (which will be further explored in another paper).

The analysis of diaries is especially suited to explore people's experiences and daily sense-making through highly tumultuous periods of crises, diffracted in many ruptures (Gillespie & Zittoun, 2010; Zittoun & Gillespie, 2012). One of its key advantages is its capacity to capture phenomena of interest on a regular basis, in context, and over time (Hyers, 2018), which is especially useful when analysing the progression of a rapidly changing societal and personal situation (such as the Covid-19 pandemic). In addition, as the present diary is collectively written, it adds to the addressivity inherent to any dairy (Lejeune, 2000) the fact that each participant reads and responds to other entries, knowing to be read. As such, a collective diary lends itself to longitudinal research of an unfolding phenomenon, which comprises different levels of analysis: it captures the microgenetic changes within and between entries, the ontogenetic changes of the diarists' identities over time, and the sociogenetic progression that frames individual experience and shapes what is possible (Douglas, 2020; Hyers, 2018; Karwowski et al., 2021; Zittoun & Gillespie, 2012; Zittoun et al., 2008). According to The Great Diary Project (North, 2012), no other kind of document offers such a wealth of information about daily life and the ups and downs of human existence.

### Analysis

The diary, initially written in Slovenian, was translated to English and imported into ATLAS.ti for the analysis. The diary entries were categorized by which week in the two-months period they were written, which serves as basis to observe the temporal unfolding of the phenomenon, exploring which codes appeared more frequently during which phase of self-isolating. This approach follows the shifting, emergent nature of sense-making processes, in which people actively reconfigure their realities through imagination and symbolic resources (see Power et al., 2023).

More specifically, we performed a content analysis (Stemler, 2001, 2015), using a mix of an empirically driven approach to identify and categorise the themes that appeared in the data, and emergent coding, using a recursive abductive movement between theoretical constructs relating to the rupture-and-transition model and the loop model of the imagination, and the indicators of these processes that were observable in the data. The codebook was first expanded to encompass 78 unique codes, and subsequently pruned to focus on the main dimensions of interest, namely a) the experience of rupture along the four main dimensions; b) the relationship between the experiences of rupture and familiarity; and c) the use of cultural resources to make sense of the situation.

The experience of rupture was operationalised as the participants’ reporting of a discontinuity or unfamiliarity. We subsequently performed a closer reading of the 180 segments coded “discontinuity” and found that the participants’ experiences of discontinuity can be categorised along four main axes, which we refer to as the four central dimensions of a rupture:

Based on the frequencies of identified codes, we used Excel to draw graphs and tables demonstrating the prevalence of codes over the period of eight weeks. Since many more entries were written in the first weeks, we used proportional instead of absolute frequencies to observe how the frequencies of specific codes changed over time, calculated with the formula *absolute frequency/number of words* × *10000* for each of the eight weeks. We use the graphs not for the purposes of inferential statistics, but as a tool to elucidate general trends in the data, explore the unfolding of the phenomena over time, visually depict the developments observed in the data, and condense 164 pages into a more content-based analysis that lends greater validity to the close, zoomed-in analysis of selected extracts (see Gillespie et al., 2024).

Finally, we identified traces of imagination in the data as distanciation from the participants’ present circumstances, as engagement with distal spheres of experience (e.g., symbolic mobility, imaginatively travelling to real or fictional places), located in remote temporalities (constructed futures, pasts, and counter-factual presents), as well as evoking absent others (such as family members abroad or constructing the motives of politicians; Glăveanu & de Saint Laurent, 2015; Grossen & Salazar Orvig, 2011), drawing on cultural artefacts (e.g., making sense of experiences via films and literature), social representations and discourses (Zittoun & Cerchia, 2013; Zittoun & Gillespie, 2016). In addition, multiple levels of imaginative work appear in the data; some entries elucidate dialogic imagination, the diary and others’ entries becoming resources for imagining, such as when writing about the news leads the diarist to wonder about possible futures; second, participants often reference past imaginings, such as their experiences while watching a film or playing a board game; and third, the process of writing a diary entry itself, reflecting on the experiences and weaving them in a meaningful narrative, largely depends on a form of narrative imagination (Bruner, 2003); especially this last level is implicit and often cannot be observed.

## Results

In the rest of this paper, we will first present the context in which the collective diary was written, namely the first wave of the pandemic in Slovenia, followed by the presentation of the quantitative results based on the content analysis of the entries. This will be succeeded by a more detailed qualitative analysis, which will combine a transversal overview of the three distinct phases of the rupture and familiarisation with an analysis of representative extracts from the diary, which exemplify the characteristics of each phase.

### Context: The First Wave of COVID-19 Pandemic in Slovenia

The first case of COVID-19 infection in Slovenia was confirmed on March 4th 2020, and the government declared a state of a pandemic on March 12th, when the number of cases in the country reached 96. Over March, progressively restrictive measures were implemented (see the timeline in Table 1), with the beginning of the so-called lockdown, or quarantining period, on March 20th. Public events, social gatherings, non-essential commercial and sports activities were suspended, and education moved online. Social distancing and wearing masks in closed public spaces were mandatory, and mobility was confined to municipalities. The end of the first wave of the pandemic was declared on May 14th, after a series of loosening restrictive measures. The outcome of the first wave of the pandemic, with a relatively low total number of cases (0.07% of the population) and 109 deaths (0.005% of the population), was generally considered a modest success (Cerar, 2020). However, it was a politically tumultuous period, since on March 13th, a new government coalition of right-wing parties under the leadership of Janez Janša took over the moderate-left coalition after the former Prime Minister, Marjan Šarec, resigned in January. The new government was met with a great deal of opposition from the people, who were critical of its handling of the COVID-19 pandemic and felt that the Prime Minister was using the state of the crisis to implement unlawful policies (Čufar & Hawlina, 2025; Delić, 2020). The trust in the government was low (Božič & M.V., 2020; Potič, 2020), there were many criticisms of the government’s actions and communication about the virus from the medical community, revelations about corruption in purchasing masks and ventilators, and researchers observed a spread of conspiracy theories and misinformation (Hočevar Grom et al., 2021). The general distrust in the official handling of the crisis contributed to the sense of uncertainty and anxiety about the situation, and due to the conflicting sources of information, people (including our participants) did not feel that they had reliable cultural resources to reconstruct a stable set of meanings. In May, as the restrictive measures were being lifted, many people took to the streets in bicycle protests against Janša’s government (Potič, 2020), including many diarists.

**Table 1:** Operationalisation of the four dimensions of ruptures

Dimension	Indications
Temporal	Participants express a sense of discontinuity with the past and interrupted futures, a disrupted sense of time, or living in an unprecedented, exceptional time (e.g., “In the spirit of exceptional circumstances, […]”, “The days are merging together, I feel like I’m reliving the same day over and over, Groundhog Day-style.”)
Spatial	Indications of disrupted relationship to space, reacting to restrictions of movement, emerging new significations of one’s home as an office or a prison, a sense that foreign countries are suddenly farther away, etc. (e.g., “It’s very hard, because I can’t go out for a walk. I’m used to taking a bus to the cemetery and walking back every day.”, “The streets are empty and it feels like there’s a holiday in the city, like everyone left on a trip.”, “Everything is empty and even the weather is post-apocalyptic. Signs everywhere warn you to stay at home.”)
Social	Changes in relating to others, both the physically present others, the familiar others, and the mediated, generalised others (e.g., “Self-isolation – what an interesting expression for a state in which you are alone at home, and yet these days, I feel more connected to humanity than ever before, since we’re bonded by a shared crisis.”, “We are afraid of each other.”)
Embodied	Indication of disrupted relationship to one’s body, the reimagining of one’s body and its presentation, a sense of disrupted biorhythm and routines, somatic indicators of anxiety, etc. (e.g., “I’ve spent an uncommonly long time pondering whether to shave my face. Given that I’m working from home next week, I decided to postpone it.”, “It was a very lame day, I was battling cramps the whole time because I read that you shouldn’t take ibuprofen now, since it can drastically worsen your state if you get the virus.”)

### Quantitative Analysis of the Diary Entries

**Fig. 1:**
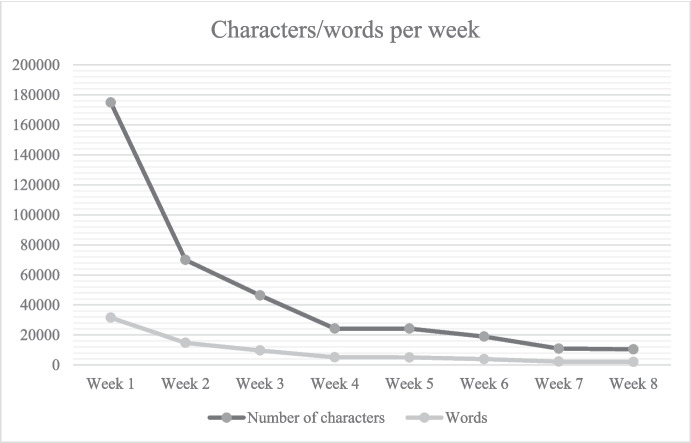
The total number of characters and words per week

A first indication of the work of meaning-making in which the diarists engaged can be seen in the actual activity of writing. Interestingly, people wrote unequally during the confinement, with a clear peak in the first week. Figure 1 shows the number of words and characters that the participants wrote over the first wave of the pandemic. During the first week, participants wrote more than twice as much as during the second week, and subsequently, the number of entries continued to decline. Since the participants used the diary as a tool for personal and collaborative meaning-making of the pandemic-induced rupture, and to reflect (as well as document) a highly unfamiliar period in their lives, we propose that when there is more disruption in the habitual meanings and practices, and the rupture is experienced more acutely, there is more activity of sense-making, in this case expressed through writing a diary. Similar observations have been made in the case of diaries written during the Second World War, collected for the Mass-Observation Archive (Zittoun et al., 2008); as the unfamiliarity became less novel and more normalised through semiotic elaboration and the use of symbolic resources (Zittoun et al., 2003, 2021), the need to write down the experiences every day was diminished, and the entries were less abundant. Hence, we can think of time as an indicator of the meaning being made, and the words written as indicators of the intensity of the meaning-making process.

**Fig. 2:**
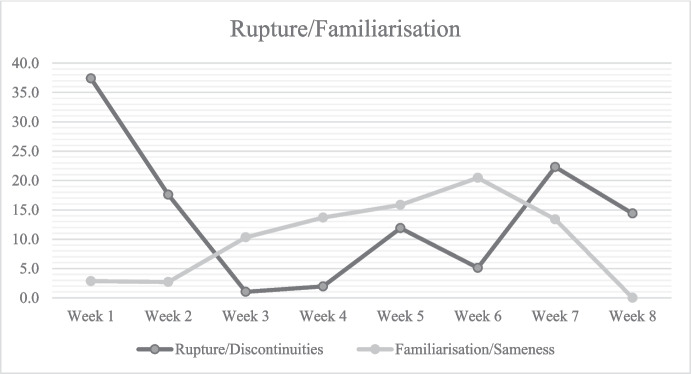
The proportional frequency of indicators of rupture and familiarisation

If the diary functions as means for meaning-making, then we can hypothesise that the experience of rupture fades with time, as people have made sense of it. To support this hypothesis, we followed indications of “discontinuity” and its evolution over time. In Fig. 2, discontinuity is an operationalisation of indicators of the experience of a rupture (see also Table 2). Sameness is an indicator of the experience of (occasionally exasperating) familiarity. We can observe three distinct periods across the span of the two months; a) the beginning of the pandemic, characterised by a strong sense of a rupture, b) the adaptation and familiarisation during the time in self-isolation, which becomes “the new normal”, and c) the mixture of familiar and unfamiliar as the measures began to loosen and people could re-engage with the outside world and the others (see the timeline in Fig. 1).

**Table 2:** The timeline of the first wave of COVID-19 pandemic in Slovenia

Date (2020)	Event / News / Situation
4th March	The first case of COVID-19 infection in Slovenia is confirmed
7th March	Restrictive measure: the government restricts public gatherings to 500 people
9th March	Restrictive measure: the government restricts public gatherings to 100 people Higher education begins suspending classes in large auditoriums and certain study programmes
10th March	Restrictive measure: All flights from Italy, South Korea, Iran, and China are suspended. Introduction of stricter border controls
12th March	Slovenian Government declares the state of epidemic
13th March	A new government coalition of right-wing parties under the leadership of Janez Janša takes over the moderate-left coalition of Marjan Šarec
14th March	The first confirmed case of death as the cause of COVID-19
15th March	Restrictive measure: The closure of all restaurants, bars, cafés
16th March	Restrictive measures: The closure of all shops selling non-essential goods Kindergartens are temporarily closed, and institutions of primary and secondary education move their teaching online Public transport is suspended
17th March	Restrictive measure: All flights are cancelled (except for humanitarian or medical purposes)
18th March	There is a critical shortage of face masks. 1,5 million of them were reportedly held up in transition
20th March	Restrictive measures: De facto quarantine is established. There is a ban on all social gatherings (with the exception of households and families), movement in public spaces is restricted
24th March	The government confirms an urgent corona package/assembly of measures (mostly monetary support) to ease the consequences of the epidemics for citizens and enterprises
30th March	Restrictive measures: A ban on travel between municipalities Mandatory use of masks and gloves in closed public spaces
6th April	The number of recorded cases surpasses 1000
28th April	The opening of public libraries, galleries, and museums
1st May	The first bicycle protests before the Parliament building in Ljubljana
2th May	For the first time since the beginning of the epidemic, there are no new confirmed cases
4th May	The opening of the restaurant services outside (on terraces and gardens), opening of hair and cosmetic salons
5th May	The opening of travel between municipalities
8th May	The second bicycle protests before the Parliament building in Ljubljana, and in several other Slovenian cities
14th May	The government declares the end of the epidemic state in Slovenia The measures that remain are the ban on the gatherings of more than 50 people and the mandatory period of self-isolation upon entering the country

In the first two weeks, participants experienced the situation as extremely unfamiliar, and there were many discontinuities on different levels (personal routines, prohibited social gatherings, new regulations, behavioural prescriptions, etc.). However, through the weeks in self-isolation, the participants experienced the situation as increasingly, often even stiflingly familiar as there was a pronounced lack of novelty and the possibility of diverse experiences. When the preventive measures began to relax in the last two weeks, people experienced a second, smaller rupture from their new stage of adapting to the pandemic situation (“the new normal”); they then felt the return to “the old normal” as slightly destabilising.

Hence, this first quantitative reading first confirms that the pandemic, experienced as confinement, was lived as rupture by the participants, creating a sense of discontinuity from their taken-for-granted lives. It also suggests that with time, and perhaps through the activity of diary writing, the sense of unfamiliarity soon fade out, and that a new stabilisation took some time to be acquired. But what sort of rupture was actually experienced? We then tried to further characterize it along the four dimensions that we identified.

As we can see in Table 3 above, during the first two weeks, the pandemic situation was perceived as the most unfamiliar and destabilising along all of the four dimensions. In the last two weeks, the return to a state that bore a closer resemblance to a pre-pandemic state was also perceived as a discontinuity from a “new normalcy” that was established during the period of self-isolating – the discontinuities were mostly along the dimensions of sociality and spatiality, since the restrictive measures relaxed people were able to move and meet freely once more. During the middle phase of self-isolating (Week 3–6), there were fewer indicators of an experience of a rupture, as people grew habituated to the conditions of the confinement. Overall, the most commonly experienced dimension of rupture was sociality, especially in the first week, while the changed sense of the embodied self was mostly evident in the first two weeks. Participants reported a heightened awareness of the body as a vulnerable site of possible infection, and different measures they took to protect it. Many participants reported an embodied sensation of anxiety (in their stomach, behind their eyes, etc.), and interpreted any cough, sneeze, or tiredness as possible symptoms of a COVID-19 infection.

**Table 3:** The frequencies of the experience of the four dimensions of rupture over time

	Week 1	Week 2	Week 3	Week 4	Week 5	Week 6	Week 7	Week 8	Total
Embodiment	24	5	0	1	0	0	0	0	29
Sociality	50	13	0	0	1	1	3	1	69
Spatiality	27	8	1	1	2	1	5	3	48
Temporality	34	6	1	0	3	0	0	1	44

Hence, we have established that people experienced ruptures due to the collective crises of the pandemic, and that with time, they managed to make sense of them, restoring a sense of continuity and normality. But how did they accomplish that, what were the meaning-making processes involved, and what was the role of the imagination?

### Qualitative Analysis of the Progression of the Rupture and Familiarisation

In what follows, we now propose to qualitatively retrace the work of meaning-making of the experienced ruptures, both externalised and enabled by the collective diary. The diary offers unique longitudinal access to these transformations; following the three phases identified in the quantitative analysis, we show the processes of sense-making and imagination along the four dimensions of rupture.

#### Weeks 1 – 2: An Acute Sense of a Rupture

The pandemic and the beginning of self-isolating was perceived as a rupture on multiple levels. On the temporal level, people experienced a disruption of normal habits and practices (personal temporal organisation), as well as grappled with the loss of collective events, rituals, and institutional routines (e.g., "I'm losing the sense of time, the days are merging together. I feel like I keep repeating the same day. Groundhog Day or whatever. I don't like it."; "Today is Easter Monday, which should be a holiday. Nevertheless, we have a Zoom work meeting at 11 h, because these days, collective time doesn't exist anymore.") (on social temporal organisation, see Adam, 2013; Lewis & Weigert, 1981). With the suspension of collective time markers, time seemed to become undifferentiated, as for many participants, there was no distinction between a workday, weekend, or a public holiday. The challenge was to establish habits and routines in self-isolation, and to create personal or private time markers to organise their days and weeks in the absence of cultural guidance on structuring time. Furthermore, participants expressed the sense that they live in an exceptional, or historical time. They often anchored the current crisis to historical events, such as wars and past epidemics (e.g., the plague, Spanish influenza), which were common pandemic narratives at the time (Chapman & Miller, 2020; Gök & Kara, 2021; Isaacs & Priesz, 2021; Oswick et al., 2020). They also projected the present moment into the future, remarking that people will look onto the pandemic as a significant historical event, and even conceptualising the diary as a future historical document. Otherwise, long-term and collective futures were perceived as uncertain and difficult to imagine; we observed an increased focus on the proximal, personal futures, such as planning the next trip to the grocery store, visiting family members, and organising household tasks. The pandemic period was perceived as temporary – participants lived in suspended time, awaiting the return to the “normal”, pre-pandemic past.

On the spatial level, the rupture was triggered by the newly imposed restrictive measures (see Fig. 1), which confined people to their homes, limited the available spheres of experience (Zittoun, 2016; Zittoun et al., 2021), and changed the rules of appropriate behaviour in public spaces. Different spheres of experience (workplace, downtime, socialising with friends and family, etc.) became conflated to the single space of one's home (e.g., “I have no other choice but to stay at home. Yesterday, I tried working from home in pyjamas, but it didn't work. Today, I needed to ritualise everything, put on proper clothes, and sit behind a computer. Because I can't go out, I can at least go out/escape on the internet.”). Like with the lack of temporal boundaries, spatial boundaries have thus dissipated, and people needed to construct symbolic boundaries for activities that were previously geographically separated.

Mandatory self-isolation also acted as a rupture along the social dimension, as people could no longer meet face-to-face. A new set of social norms was introduced in a very short time span, requiring people to maintain social distancing, wear masks and gloves, and disinfect their hands regularly. Most of the contact with friends, family, and co-workers was mediated via telephones, social networks, and video calls. Participants wrote about calling different people to check on them, to exchange information and make sense of the situation, and to pass the time by catching up with friends and playing games over videocalls. On the societal level, participants followed the news and reacted to what was happening nationally and internationally. They expressed anxiety over the political situation, overburdened healthcare systems, and solidarity with people's suffering.

In the beginning of the pandemic, the participants’ diary entries also showed discontinuities in the ways they related to their bodies. The body was perceived as fragile and vulnerable, and reimagined through the use of diverse analogies (e.g., feeling like you’re a 100 years old, like you’re hospitalised, wrapping yourself in protective gear like you’re going to Antarctica, etc.). It is interesting to note that these changes experienced in the body were spontaneously expressed via the imagination of a distant time and place, constructed using fictional resources.

More generally, during the first two weeks, symbolic resources (Zittoun, 2021; Zittoun et al., 2003) were often used to make sense of the situation, drawing on elements from films and novels (e.g., *Jason Bourne* for feeling like being in an action movie, *Monty Python* for the absurdity of the political situation, *Groundhog Day* for the endless repetition, red shoes from *The Wizard of Oz* for imagining teleporting oneself elsewhere), and creatively recombining them to apply them to their current experience. Films, television series, books and videogames were employed both to actively deal with the rupture and make the unfamiliar situation less uncertain, as well as to distanciate from the uncertainty and anxiety of the pandemic, finding comfort and sometimes refuge in fiction (Zittoun, 2006). Finally, cultural artefacts were also used as resources to organise and differentiate time, such as establishing a daily ritual of watching long-running TV series and rationing the available episodes, planning to organise a large music collection over a period of a month, or using a material artefact such as a calendar to represent and organise time in a very concrete way (physically crossing out days, anticipating the future).

*Example 1: Ada, March 15th*

It is very unusual for me to travel to Ljubljana and not immediately call a friend for a movie and coffee. The family council rejected my idea of a “Decameron party” where 7 girls and 3 boys would tell each other stories. Now all I have left is Netflix. I talked to Ana about the situation among doctors, it is very difficult to create any image of the future. I no longer know the world and I have no idea how long such a state of emergency will last. Will our normal past become completely extraordinary? For now, I wish everyone well and hope that the healthcare system doesn’t collapse and that Janša [PM] does not privatize it.

Oh, and as for coping music: YouTube suggested *Apocalypse from Cigarettes After Sex*, it makes me very nervous – the lyrics go “your lips, my lips, apocalypse” and I scream “Go apart and keep a social distance”! Irresponsible youth.

There are many things to observe in this entry in terms of experiencing a rupture, temporal movements between the past and future, and experiencing the situation through symbolic resources. Ada writes about experiencing a rupture along different dimensions: she begins with the social dimension, by saying that her usual habit of meeting up with friends has become inaccessible, and that her family counselled against organising a themed party. From there, her writing moves to a rupture along the temporal dimension, namely the inability to imagine a future and an overwhelming uncertainty. The discontinuity in her experience of time is best captured by her question “Will our normal past become completely extraordinary?”, showing a qualitative shift between a “normal”, pre-pandemic past and the pandemic present, from the position of which the past is recontextualised as “extraordinary”. This closely corresponds to Mead’s (1932/1980) conceptualisation of emergent events, which are characterised by a disrupted present in which a new coherence hasn’t yet been established; from this unstable vantage point, the expected future suddenly becomes a past future, and a past present is replaced by a present present without any clear future or a fixed path forward. During the pandemic, the past was reimagined not as continuous with the present, but as a qualitatively different state before the advent of the SARS-CoV-2 virus, the present was uncertain and needed to be made meaningful and comprehensible, and while the old futures (such as travel plans, social rituals, and career agendas) suddenly became impossible, people struggled to construct new plausible futures and re-engage with imagining their lives extended in time.

We can observe the movements of the imagination along different axes in the active attempts to make sense of the unfamiliar situation. As mentioned, Ada attempts to make sense of the present with an imaginary, counter-factual movement “Will our normal past become completely extraordinary?” (Byrne, 2005). In addition to imagining in time, she uses a variety of symbolic resources. She relates the present pandemic to the historical fourteenth century plague encapsulated in Giovanni Boccaccio’s *The Decameron*, a story about a hundred tales told by a group of ten young people while they isolate themselves in a secluded villa to escape the plague. It enables her to imagine organising a “Decameron party”, where ten contemporary young people would tell each other tales to cope with the period of social isolating, evoking the potential of storytelling to get through crisis periods. Because that option is invalidated, she then concludes “Now all I have left is Netflix.”, suggesting that when imagining with others is inaccessible, she has recourse to a technologically mediated platform that enables the distanciation from a ruptured present into fiction – culturally guided imagination. The participant then engages in a recursive looping between the present, the future, and the past, trying (and failing) to obtain information that could act as material to construct imagined future scenarios. She reflects on the past and expresses a modest hope for the future as it pertains to the healthcare system, which corresponds to the present crisis and a lack of trust in the Prime Minister and his motives. Finally, Ada refers to a platform (YouTube) for accessing symbolic resources, through which the participant makes sense of a disrupted, apocalyptic present. She interprets the romantic lyrics “your lips, my lips, apocalypse” in relation to the embodied disruption; bodies now become dangerous, and the lyrics seem to trigger the imagination of a pandemic “apocalypse” – which leads her to distance herself from other “irresponsible” youth.

Hence, in this sequence, the participant engages in sense-making of the rupture experienced spatially, socially, temporally, and bodily by an intense work of imagination: it is because she cannot meet friends in a café that she uses symbolic resources to imagine alternative social gatherings; the management of access to imaginary experiences via TV series enables her to reorganise time; and a song offers an imaginary way to express bodily fears.

#### Weeks 4 – 6: A Stifling Sameness

After a couple of weeks of self-isolating, the novelty of the pandemic wore off, and the boredom and ennui set in. The lack of diversity of experiences, settings, and activities, as well as a lack of socially imposed temporal differentiation made people feel like they were losing a sense of time and that days were melting one into the other (e.g., “I have a feeling, that every day from now on will be the same. What else can happen in isolation?”). People were increasingly entrenched in their routines, and the new state has become (overly) habitual – there was less anxiety and uncertainty, but the limited scope of the everyday was deeply uncomfortable. The discomfort was also experienced physically, as several participants reported feeling fatigued, “foggy-brained”, and having disrupted sleep schedules. Furthermore, some reported that their memory was becoming unreliable, or that they couldn’t remember the recent past, since the activities were so undifferentiated and almost all of them took place in the proximal sphere of one’s home. There was a growing sense of frustration regarding how long this liminal state would last, and that it was impossible to plan anything beyond the short-term future.

The number of daily diary entries began to fall, and people wrote that they have nothing to report, that their days are empty or the same as the ones before. The entries sometimes became less related to what was happening in the present, and more general reflections on the period, the past and the future, as well as meta-reflections on the writing of the collective diary.

In weeks 4–6, participants used cultural elements as symbolic resources to make sense of their experiences, and to communicate the unfamiliar feelings. The diary itself was becoming an increasingly important resource for organising time, an aid for remembering, and maintaining a sense of connection to the others. Participants reported feeling more and more confined in their homes, municipalities, and within national borders. In this vein, symbolic resources were commonly employed to break away from the confines of one’s proximal zone of experience and exploring distal spheres of experience. In some cases, the distal spheres related to real places, such as the imaginings of travelling that is impossible under the restrictive measures (symbolic mobility); in some cases, participants travelled in time instead of space, either nostalgically remembering the past or trying to envision a post-pandemic future; and sometimes, the distal spheres of experience were entirely fictional, such as when people disengaged from the present by watching films, television, or reading novels.

*Example 2: Sandra, March 29th*

It’s already 8pm...?? Today the clock has moved [daylight savings time] and I feel like Alice [in Wonderland]. Or even better, the White Rabbit from Alice, who just rushes around with that gigantic pocket watch (I don’t know if we all had the same illustrated book?) and is late, late, late. To me, it’s all day today in his style.

The extract above is very demonstrative of the experience of time in the period between Week 3 and Week 6, when the initial period of heightened rupture and uncertainty has passed, and people are dealing with the almost overwhelming familiarity of confining themselves at home. In the absence of collective time markers, the participant describes losing a sense of time, and she relates this experience to the character of the White Rabbit from the novel Alice in Wonderland, using a symbolic resource to make sense of her feeling of simultaneously being in a hurry and late. We also see an element of the addressivity in the collective diary when she poses the question whether the participants all had the same illustrated book, thus checking if they can relate to how she imagines herself.

*Example 3 Tina, March 30th*

The hardest part about isolation for me is that we don’t know when it will all end. I understand why we don’t know, but it eats at me anyway. Because I just like to plan things and I’m not infinitely spontaneous. Can’t they tell us “by the end of May, and that’s it.” Until July, until September, I don’t care, I just want to know. Unrest is already in the air from all this isolation, and the worst is yet to come as far as the corona is concerned. Unrest is in the air – that’s how a Hollywood trailer for this apocalyptic action movie would start. Unrest is in the air, people are leaving their homes and John Smith only has a week left to save humanity: from himself and from the deadly virus! The main role would of course be played by Keanu Reeves.

In this entry, we once again observe the theme of a disrupted relationship to time, in this case brought about by the participant’s inability to imagine the future. She is anxious to know until when the pandemic self-isolating situation will last, and in the absence of a clear collective guidance (in this case the government’s lack of communication regarding the strategy and the prolongation of preventive measures) blocks her ability to project herself forward in time, which “eats at her” in the present. In this sense, the overwhelming uncertainty acts as a barrier to the imagination, and the participant cannot repair the temporal rupture. However, she reframes her experience through another movement of the imagination, using the narrative template of Hollywood action films to make sense of the anxiety and unrest in the “apocalyptic” situation of the pandemic.

*Example 4: Ada, April 5th*

The days are still similar, almost the same, this week I felt tired all the time. As with those sensory deprivation experiments, I find myself becoming dense, stupid, tired due to a lack of diversity and novelty. […] I would love to go to sea, who knows when they will open the borders.

In the evening I tried to write a book, but it went slowly and painstakingly. […] At the start of the pandemic, I was thrilled, quarantine is ideal for novelists! Now, however, hardly anything comes out of me.

Above is another instance of a participant describing the oppressive sameness and the lack of diverse experiences during the self-isolation. Here the temporal rupture has further consequences: the lack of mental stimulation is experienced physically, as sense of fatigue, which Ada relates to the sensory deprivation experiments. The spatial disruption brings her to express a desire for geographic mobility (going to the sea). Yet again she invokes the uncertainty engendered by the lack of a clear strategy on behalf of the Slovenian government (“who knows when *they* will open the borders”). Being confined to a single place and unable to project herself in time also acts as a hindrance to the imagination; she expresses the frustration that she is struggling with creative writing, especially since at the beginning of the pandemic, she thought it would be an ideal time for it. If we make a connection to her entry from March 15th, there is a thread of *The Decameron* and the notion of passing the pandemic by telling stories. However, we can see that self-isolation, uncertainty, and the lack of diverse experiences that could nourish the imagination can also be detrimental to it.

Hence, after the first effect of the rupture experienced by people, they now seem to enter a suspended time, which also has consequences socially, spatially and physically. People know when the rupture creating a special time started, yet cannot know when it will finish – similar to the experience of “exceptional times” during a war, for instance (Zittoun & Gillespie, 2015). People start to suffer from the uncertain ending (see Examples 3 and 4).

The pandemic thus appears as liminal experience, the middle phase of any ritual or collective transition, characterized by the fact that it is an “in-between” state of the world with its particular social and temporal structure, mediating between an old and a new status quo (Cangià, 2021; Stenner, 2018, 2022). However, not having an announced ending, the pandemic was at risk to become a liminal hotspot – a state of being stuck “in the zone” (Stenner, 2020). All depends on how things will evolve, and whether a possible end to the liminality can be imagined. In particular, art and narrative structures can be crucial in helping people structure otherwise ambiguous or fragmented experiences and navigate prolonged uncertainty (Grabska & Horst, 2022).

#### Weeks 7 – 8: Re-entering the World

Towards the end of April and beginning of May, the number of daily new cases of COVID-19 infections was steadily very low, and the government began to loosen the restrictive measures (Table 2). Public spaces, cultural institutions, and municipality borders were beginning to open, and people felt like the pandemic period was coming to an end.

Expressions of discontinuities (see Fig. 2) became more common in the diary entries, as people experienced the return to a tentative “normal” state as a departure from the pandemic state, leaving behind most of the habits and routines that they have formed during self-isolation. There was the sense that the participants were departing from the liminal state of suspension in time and space, and re-joining the society and external world beyond the confines of their homes. Consequently, the number of entries continued to fall (Fig. 1), and there was less of an urgency to make sense of an unfamiliar situation, and more of a desire to reflect back on the period of self-isolating. Participants narrated the progression of events from the beginning of the first wave of the pandemic to its ostensible end, and took stock of what they have accomplished and experienced in self-isolation.

Beyond the focus on the proximal sphere of experience, the immediate present and the short-term future, participants were re-engaging with the collective past and opening up possibilities for personal and collective futures. Public events were back on the agenda and as many people returned to work, they regained the socially imposed structure of time. An important event that united many participants were the bicycle protests, which started to take place every Friday in front of the Parliament building in Ljubljana (M. P. & M. B., 2020). Many people wrote about how wonderful it was to see familiar and unfamiliar faces in person, and the joy of reuniting with friends and family they haven’t seen in weeks or months.

In the last two weeks, symbolic resources were used to connect the present to the past and the future and reimagined themselves as part of the community, which generated a sense of optimism and hope for positive social change. Several participants invoked the Yugoslav past to criticise the present, but also to express hope for a future with greater solidarity and sense of connection. Because geographical mobility was possible again, people were re-discovering the world and expressing the freedom of crossing borders and leaving the confines of the home. There were no more cases of symbolic mobility and using cultural artefacts to escape the pandemic situation; instead, books, films, and poems were sometimes used to make the time of self-isolating more concrete and quantifiable, or to express resistance and hope for the future.

*Example 5: Nada, April 28th*

Measures are being lessened and our daily Notes are also lessened. These were most numerous at the beginning of self-isolation, at a time when we did not know quite well what was happening to the world and what was happening to us, what to fear, what was approaching us, how to position ourselves. Visiting a shop or a pharmacy has already become a daring adventure and the main event of the week (remember?), and we went for small walks as frightened illegals of occupied Ljubljana (when was that already?). Then, after all the baked pancakes and muffins, over-saturated news with anointed Kacin [minister of health] on the forefront, too long and too short nights, we each got used to our own way of self-isolation. The days became more and more similar and began to merge into a strange mixture of micro events, new habits, unexpected insights, missing things, restlessness and peace, a lot of wasted time, precious fermentation and waiting for… ..to what?

All indications are that we are now, after almost six weeks, on our way back to “normalcy”. How we will walk, walk or tread this path, what known-unknown environment we will enter, what will remain the same and what is gone forever, we will see and feel on our own and foreign bodies in the coming days, weeks and months. We are a part, a small part, of something bigger.

Nada reflects on the experience of self-isolation, describing the different phases of its progression: the acute rupture accompanied by fear and uncertainty, the stabilisation into routines and a sense of similarity from one day to the next, and finally, the return to an ambivalent, open-ended “normalcy”. Nada describes the initial period of heightened, collective meaning-making, connecting the large number of written entries to the shared uncertainty and disruption of habitual meanings. Via an imagination of the past, she connects the frightening experience of the highly regulated movement through the city to the historical context of the occupation of Ljubljana during the Second World War (1942—1945) and uses the concreteness and regularity of food and news as markers of the time that has passed. The reflection on the weeks in self-isolation ends on the difficulty of imagining the future. Re-entering the world and the return to an ambivalent “normalcy” present a new kind of rupture, and the participant expresses the need to stabilise the old meanings and create new ones in the face of an uncertain future.

### Imagination along Four Dimensions of Ruptures

In terms of our analysis, as other researchers have observed in the case of economic crises (Clifford Pedersen, 2025; Power et al., 2020), societal crises do not directly correspond to people’s experience of a rupture. Hence, instead of focusing only on the representational level of psychological responses to crisis (Caillaud, 2016; de Rosa et al., 2021; Galli et al., 2014; O’Connor, 2012), we found it fruitful to explore the experiential level. In lieu of treating ruptures as a unitary phenomenon, we have thus proposed a more differentiated understanding of the experience of rupture, identifying four central dimensions along which the rupture (triggered by the pandemic crisis) was experienced. These axes are sites of both disruption and turmoil, but also of generative tensions with the potential for meaning-making, imagination, and transformation.

Imagination, which we see as the core process supporting sense-making of the rupture, thus unfolded along these four dimensions. On the temporal axis, the two central functions of the imagination were, first, to organise a sense of time in the absence of collective temporal markers, and second, to reengage with the past and the future in a time when especially the futures were completely destabilised. Second, on the spatial dimension created by the confinement, the imagination was a crucial process that enabled reaching beyond the boundaries of one's home and symbolically escaping the confinement. This was primarily achieved through symbolic resources, which afforded the distanciation by journeying into fiction – novels, TV, films, videogames, etc. Furthermore, people engaged in symbolic mobility (i.e., imaginatively travelling to physically inaccessible places, retaining a sense of belonging to a world, keeping track with what is happening abroad, and imagining oneself in distant cities; Pedersen & Zittoun, 2022; Zittoun, 2020b). Third, in terms of the social rupture, during the exponential spread of SARS-CoV-2, we saw attempts to create shared stories and interpretations of the pandemic from politicians, journalists, scientists, etc. (De Rosa & Mannarini, 2020; Stenner, 2022), as well as a more decentralised dissemination of people’s perspectives on Covid through social media – for instance, researchers have observed an increase in the creation and circulation of memes during the COVID-19 pandemic (Priyadarshini et al., 2022; Skórka et al., 2022). Finally, the COVID-19 threatened physical integrity, creating a rupture in people’s sense of embodiment. In the beginning of the pandemic, the participants' diary entries show discontinuities in how they related to their physicality. The body was made sense of through the imagination, and the use of diverse analogies (e.g., feeling like you're 100 years old, like you're hospitalised, wrapping yourself in protective gear like you're going to Antarctica, etc.), and perceived as fragile and vulnerable. In the following weeks, the body also became a physical marker of time (growing a beard, gaining kilograms in quarantine, etc.). In this multidimensional perspective on rupture, imagination emerges as a dynamic process that mediates individual adaptation and reshapes collective experiences, facilitating both personal resilience and broader cultural reconfigurations in crises.

## Discussion: Imagining Collectively Through the Pandemic Crises

In this paper, we have examined the first wave of the pandemic as a crisis, experienced as a series of ruptures by a group of people in Slovenia via their collective diary. We have furthered the theoretisations of ruptures by decomposing them into four central dimensions along which the participants experienced the discontinuities engendered by the crisis. Observing how the ruptures evolved longitudinally, we were able to chart the transformations of the imagination that enabled the diarists to cope with and creatively make sense of the unfamiliar situation and, over time, repair the ruptures along the four axes. In doing so, we hope to open new avenues of documenting crises, and to provide new ways to analyse how they are experienced, how they are made sense of, and the contributions of imagination in overcoming them.

### Sense-making and Imagining in a Collective Diary

This paper is based on the analysis of a collective diary, which was created by a group of people as a means to deal with the crises; it only secondarily became research material. It is thus interesting to note how these *Notes from Self-Isolation* enabled the diarists to express how the crisis was experienced as rupture, to narrate the events in their life, to make sense of their experience, reflect upon it, transform its meanings and valence, and imagine, connecting their day-to-day experiences with the personal past and futures and alternative realities. Additionally, the participants reported that the *Notes from Self-Isolation* provided an important memory aid in a period when time seemed suspended and there was little to differentiate one day from the next – in that sense, the act of keeping a diary helped participants to organise their experience, find something new to write about even in a time when every day seemed the same, and help them remember in an unusual period where most of the experiences took place in the private sphere of one's home. Finally, a specific function of a collective diary was that participants felt less alone in a time of self-isolation, and saw that what they perceived to be very new and unique experiences were shared with others, who were dealing similar anxieties, feelings of boredom, melancholy, creativity, etc. In addition to mutual support and solidarity that developed in the community of diary writers, the Notes from Self-Isolation took on a social function as participants shared experiences and developed written exchanges between entries, which calls for future exploration of the dialogicality in collective writing.

### The Development of Imagination over the Pandemic

To fully show how imagination supported sense-making along these four dimensions of rupture, we followed its evolution over time. By exploring the temporal progression of rupture and familiarisation, we can identify the different functions of the imagination that helped repair the rupture along the four central dimensions. Of course, these functions are not strictly independent or discrete – the same flight of the imagination can fulfil multiple functions simultaneously. We have identified three distinct phases in the process of making sense of the rupturing experience, and explored the central roles of the imagination in each one, as summarised below.

In the first phase of the pandemic, people were starting to experience the crisis as a rupture. Although the pandemic crisis was rooted in biological realities and physical restrictions, it was often *experienced as a rupture through the imagination*. First, the virus itself was imperceptible to the senses, so people imagined pathogens in the air, on their clothes, in other people’s breath, etc. In the words of De Rosa and Mannarini (2020), the SARS-CoV-2 virus became an “invisible Other”, ever-present but never directly witnessed. Second, participants reported a disconnect between their proximal situation, which often seemed very mundane, and an awareness of a crisis happening elsewhere; through the media, they imagined the global scope of the crisis and empathised with the people most affected by it. Sometimes, they described feelings of guilt when they were doing fine or even enjoying self-isolation, but were aware of the suffering of absent people beyond their proximal experience. In addition to the disconnection between the proximal and distal, and self and other, we can also observe a disconnection between the (more or less stable) present and (apocalyptic) futures; an important component of experiencing the rupture was the imagination of terrible futures, which entailed resource scarcity, autocratic regimes, and economic recession.

In the second phase, people were becoming more familiar with the disruption and started adjusting to it. Imagination had two main functions, one of *symbolic mobility*, and the second of *affective regulation*. First, it enabled people to distanciate from the here-and-now experience of the lockdown. In a period where physical bodies were highly regulated with restrictions on behaviours, socialisation, and mobility, imagination was central for enabling people’s minds to wander freely, escaping beyond the confines of self-isolation. Participants would disengage from the rupturing experience by, first imaginatively travelling in space, engaging in symbolic mobility (Cangià & Zittoun, 2020; Zittoun, 2020b), such as Tina looking at maps of New York and imagining herself walking along its streets, and what the pandemic situation is like there; second, imaginatively travelling in time, such as nostalgic remembering of visiting friends and going on road trips before the pandemic; and third, travelling from the real to fictional worlds, such as reading books, watching films and series, and playing videogames to temporarily leave the pandemic reality behind. Second, imagination was used to regulate affects (Brown & Reavey, 2015; Singer, 1976; Zittoun, 2014; Zittoun & Cabra, 2022). Imagination played an important role in helping participants manage fear and anxiety, and to rediscover hope; imaginings of possible post-pandemic futures enabled them to perceive the rupture as temporary, and gave the participants optimism that the crisis will not continue in perpetuity and that they will be able to overcome it (Pedersen, 2021). Besides positive future visions, some participants compared their situation with negative counter-factual scenarios, which made their actual circumstances seem less dire. However, the crisis did not only trigger the imagination, which would help manage the experienced rupture; in some cases, a characteristic of a rupture was also a *limited scope of the imagination*. Participants wrote that overwhelming uncertainty blocked their imagination, or made it impossible to plan for the future; a similar finding about the uncertainty limiting people’s future engagements and “life course agency” was reported by Sánchez-Mira et al. (2022). At times, our participants had insufficient or inadequate cultural resources at their disposal, hence they struggled to construct stable meanings of the situation. Other times, they reported feeling overwhelmed by the amount of information and misinformation, and needing to tune everything out, or self-impose barriers on imagining distal situations, focusing instead on the proximal where they could exercise agency. Finally, suggesting a liminal hotspot (Stenner, 2022), the lack of novelty and diverse triggers for the imagination made some participants feel like they were stuck in a mundane, never-ending present, their imagination malnourished.

Lastly, in the third period, when the end of the measures was announced, people could imagine again more freely. On the one hand, imagination became turned back as a more *reflexive movement* on the period recently lived, and on the diary itself. As in the research on the diaries written during WWII (Zittoun & Gillespie, 2022; Zittoun et al., 2008), when the acute crisis had passed, diarists began to reflect back upon it and narrativize their experiences in a tumultuous period. They attempted to concretise the time that has passed through how many books they read, how many films they watched, how many kilograms they gained, and to give meaning to the “exceptional times” from the new, temporally distant perspective (see Example 5). On the other hand, imagination was let free, and people started to *reopen their temporal and spatial imagining* – remembering the historical past to imagine the future, or imagine possible travels abroad. In the final two weeks, we observed many references to Slovenia’s socialist past in Yugoslavia and historical resistance movements (e.g., Nada and Sandra writing about the fascist occupation of Ljubljana during WWII, Petra wearing a hoodie with “Yugoslovenian” written on it because it reminds her of connectedness and solidarity, Neva lamenting that Josip Broz Tito would be disappointed with the current political situation, etc.); they were used to evoke the spirit of solidarity and resistance, which were mobilised in hopes for better post-pandemic futures. This interplay between the imaginings of the past and future was further enacted in the protest movement that began in May, as the restrictive measures were being lifted (see Table 2). The final entries are largely optimistic, as participants were joyful to see their friends and family again, and hopeful about the regained possibilities of mobility and political participation. This aligns with emerging ideas on imaginative world-making in response to societal crises, highlighting how the imagination can help construct alternative futures in times of turmoil (Bečević, 2020; Jasanoff, 2020; Jovchelovitch & Hawlina, 2018; Power et al., 2023).

### Openings

By analysing a collective diary during the first wave of the COVID-19 pandemic, this study contributes to a deeper understanding of how imagination operates as a sociocultural process in times of crisis. We have shown how imagination enables individuals and communities to navigate ruptures, make sense of uncertainty, and construct alternative futures. However, imagination – with its vital role of expanding experience when it is limited, or to regulate affects – can also be threatened in times of protracted uncertainty. In this vein, it is interesting to note that most countries officially announced the “end of the pandemic” – a significant way to collectively mark the end of a crisis, enabling people to work towards the end of their liminal experience, back to a transformed reality (Stenner & Zittoun, 2020). Altogether, we hope to have contributed to the theoretical analysis of crises as social, spatial, temporal and embodied ruptures, and the further exploration of imagination as sociocultural dynamics. Methodologically, the exploration of crises as dynamic, unfolding phenomena necessitated a process-oriented approach (Power et al., 2023) that illuminates how individuals experience ruptures, how sense-making evolves within a sociocultural context, and how people actively engage in the imaginative reconfiguration of their social realities. Rather than a passive adaptation to crisis, imagination emerges as a generative force that enables individuals and communities to navigate uncertainty, negotiate shared meanings, and create new possibilities. We also aimed to show how collective diary writing may itself become a fundamental resource for people to deal with crises and maintain hope and the imagination of possible futures. Finally, we emphasise that imagination is not merely an individual cognitive function but a deeply social and cultural practice shaped by interactions, narratives, and shared symbolic resources. As the world continues to grapple with crises, understanding the role of imagination in collective adaptation remains a critical endeavour for sociocultural psychology and beyond.

## Data Availability

No datasets were generated or analysed during the current study.

## Footnotes

1 Of course, one may question the veracity of the expression. In effect, over the last centuries, there have been other seismic ruptures on the collective and personal level, which share some characteristics of the experience of destabilisation and unfamiliarity, as well as the adaptation to it. On the collective level, the COVID-19 crisis is thus comparable to other epidemics, wars, forced displacements, natural disasters, and global financial crashes, all of which have been used as historical resources to make sense of the present situation, as shown in the studies on the metaphors people used for the pandemic (see Chapman & Miller, 2020; Gui, 2021; Isaacs & Priesz, 2021; Oswick et al., 2020; Wicke & Bolognesi, 2020).
